# Supplementary material for: Vertical organic synapse expandable to 3D crossbar array
Source: Nat Commun. 2020 Sep 14;11:4595. doi: 10.1038/s41467-020-17850-w (PMC7490352; doi:10.1038/s41467-020-17850-w)
Supplement: Supplementary file 1 — Supplementary Information [file 41467_2020_17850_MOESM1_ESM.doc]

**SUPPLEMENTARY INFORMATION**

**Vertical Organic Synapse Expandable to 3D Crossbar Array**

**Choi, Y. et al**

**Supplementary Information**

**Vertical Organic Synapse Expandable to 3D Crossbar Array**

Yongsuk Choi,^1,†^ Seyong Oh,^2,†^ Chuan Qian,^1^ Jin-Hong Park^2,3^,*, Jeong Ho Cho^1^,*

^1^Department of Chemical and Biomolecular Engineering, Yonsei University, Seoul 120-749, Republic of Korea.

^2^SKKU Advanced Institute of Nanotechnology (SAINT), ^3^Department of Electrical and Computer Engineering, Sungkyunkwan University, Suwon 16419, Republic of Korea.

^†^These authors contributed equally to this work.

*Corresponding authors

E-mail: J.-H. Park ([jhpark9@skku.edu](mailto:jhpark9@skku.edu)) and J. H. Cho ([jhcho94@yonsei.ac.kr](mailto:jhcho94@yonsei.ac.kr))

# **Supplementary Figures**


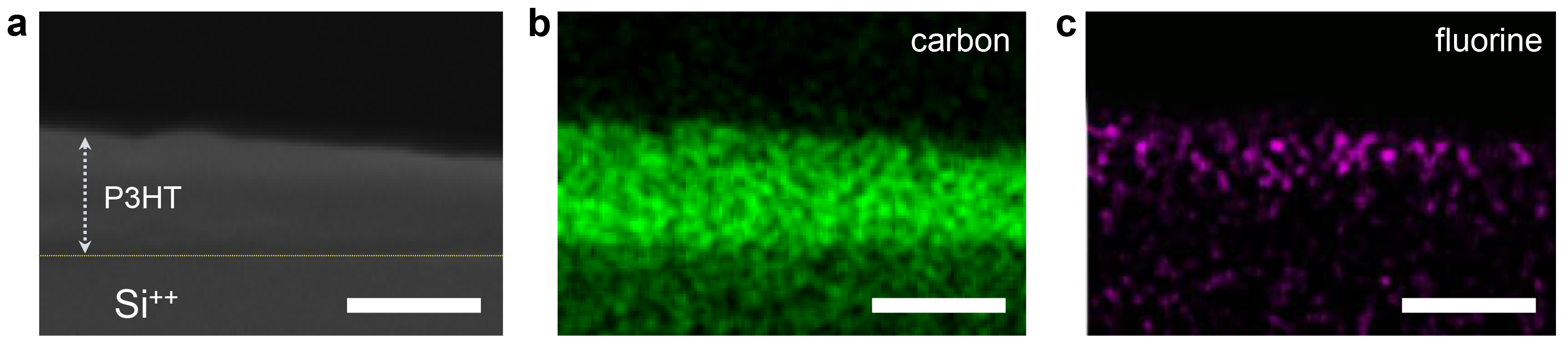


**Supplementary Figure 1 Cross-sectional SEM-EDS images of 200-nm-thick P3HT film doped with negative ions a**, Cross-sectional SEM image of P3HT film. **b-c**, Mapping images of elemental signals detected from (b) carbon in polymer chain of P3HT and (c) fluorine of TFSI^-^ ions. Scale bar is 200 nm.


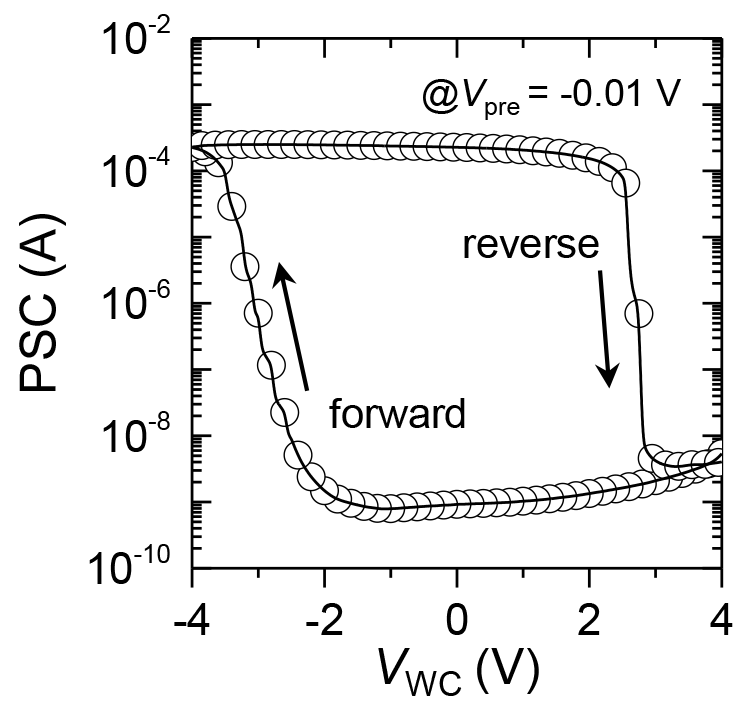


**Supplementary Figure 2 Electrical property of vertical synapse.** Typical transfer characteristics (PSC–*V*_WC_ curve) of vertical synapse. The drain voltage (*V*_pre_) was fixed at -0.01 V.


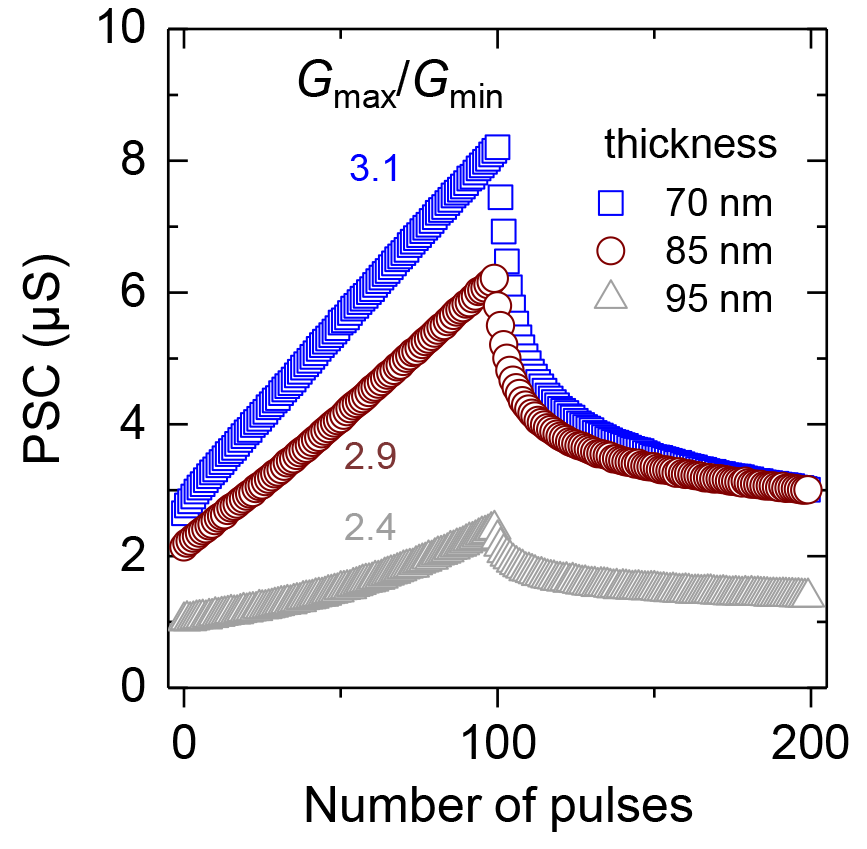


**Supplementary Figure 3 Thickness-dependent electrical property of vertical synapse.** LTP/D characteristics of vertical synapses with 70, 85 and 95-nm-thick (red circles) P3HT channels. The applied *V*_WC_ set consisted of 100 potentiation pulses (*V*_WC_ = -3 V) and 100 depression pulses (*V*_WC_ = +2 V).


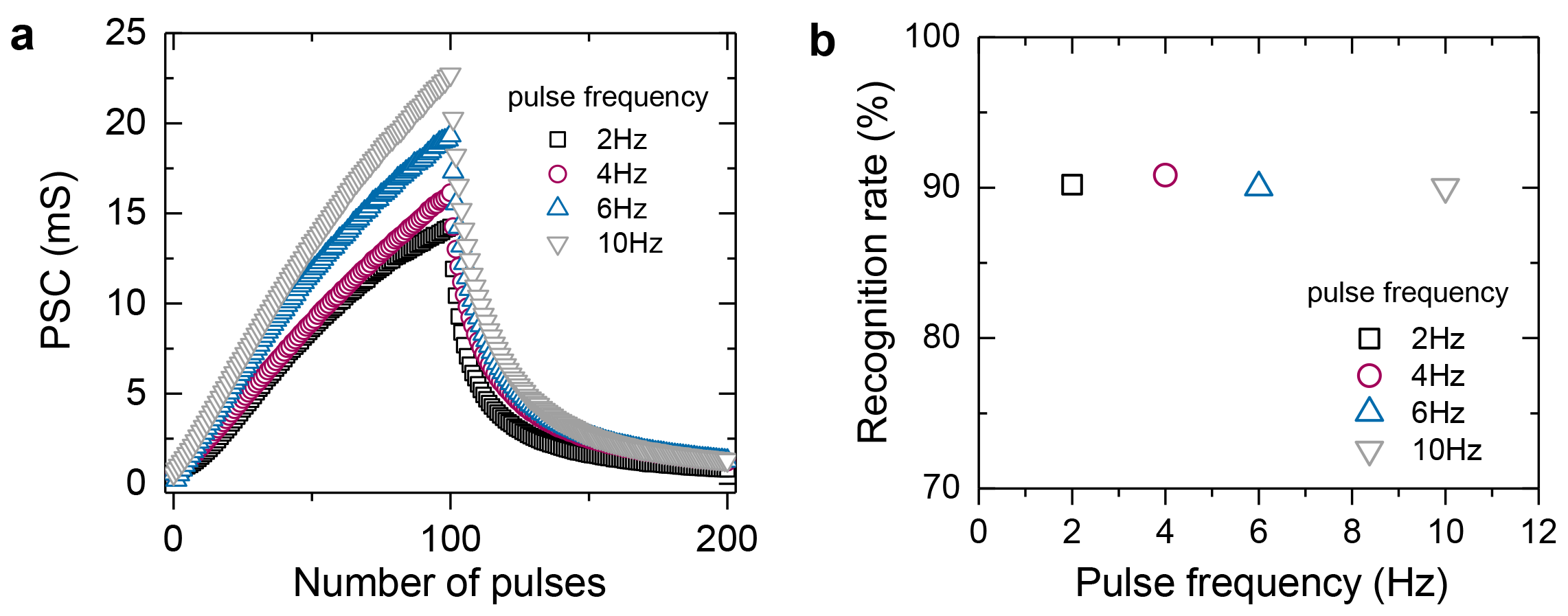


**Supplementary Figure 4** **Thickness-dependent electrical property of vertical synapse a**, LTP/D characteristic curves measured under pulse frequencies of 2, 4, 6, and 10 Hz, where *V*_LTP/D_ = −3 V/+2 V. **b**, Recognition rates with respect to pulse frequency.


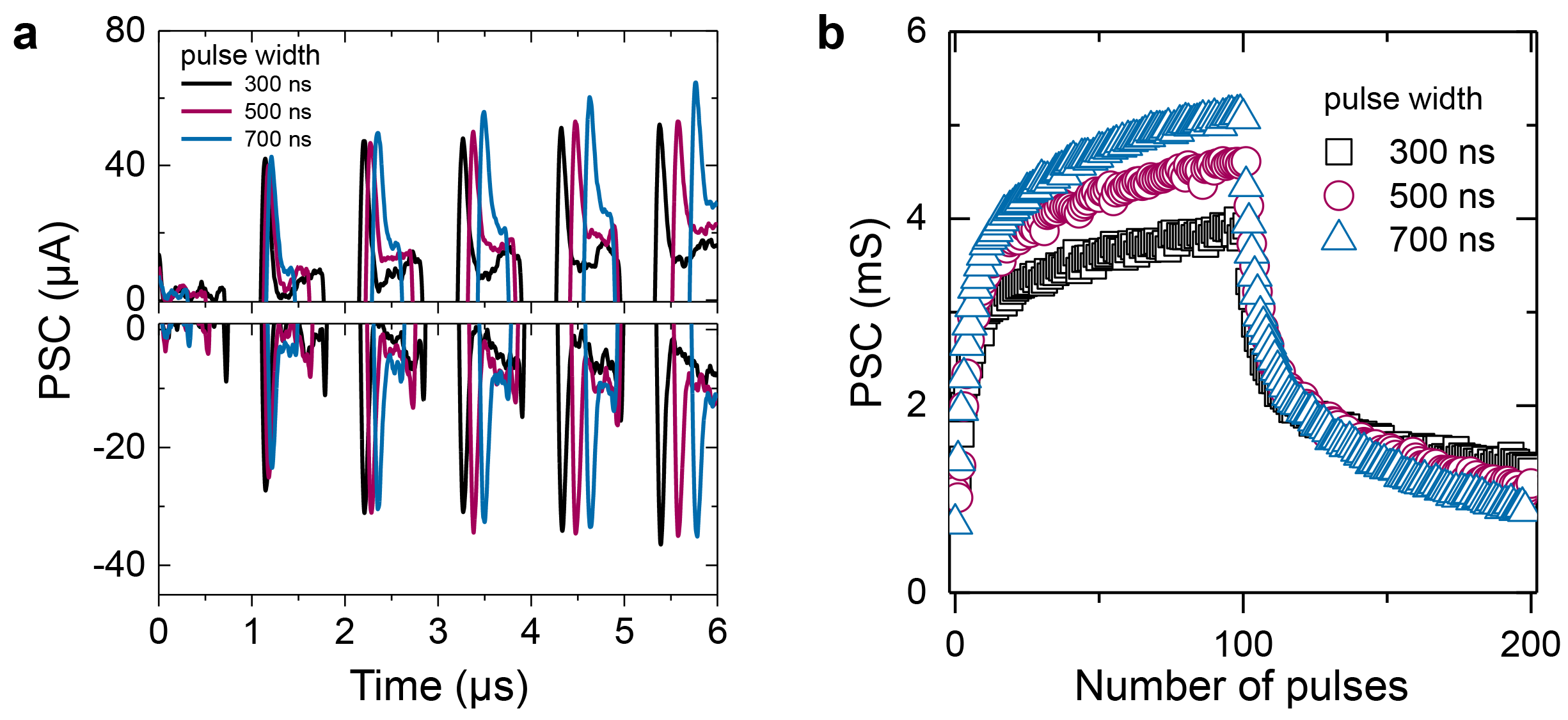


**Supplementary Figure 5 Pulse width-dependent electrical property of vertical synapse a,** PSC responses with respect to potentiation (upper panel, *V*_WC_ = −3 V) and depression (lower panel, *V*_WC_ = +2 V) voltage pulses with pulse widths of 300, 500, and 700 ns. **b**, LTP/D characteristics measured under application of 100/100 potentiation/depression pulses (*V*_WC_ = −3 V/+2 V) with pulse widths of 300, 500, and 700 ns.


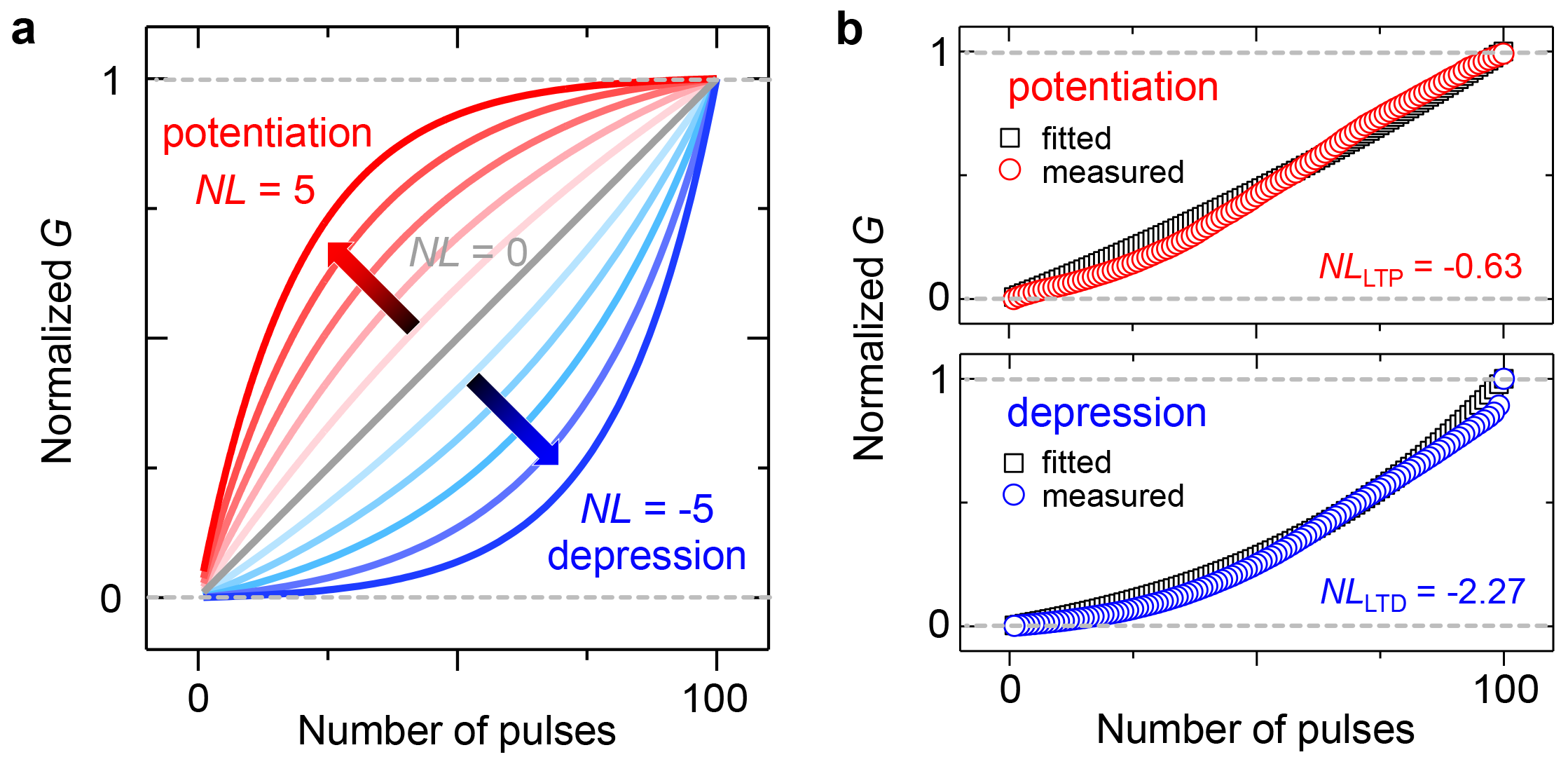


**Supplementary Figure 6 *NL* analysis of LTP and LTP characteristic curves a**, *G*_LTP_/*G*_LTD_ curves with respect to *NL* ranging from 0 to 5. **b**, Measured/fitted curves in LTP (upper panel) and LTD (lower panel) regions, where *NL*_LTP/D_ = −0.63/−2.27.


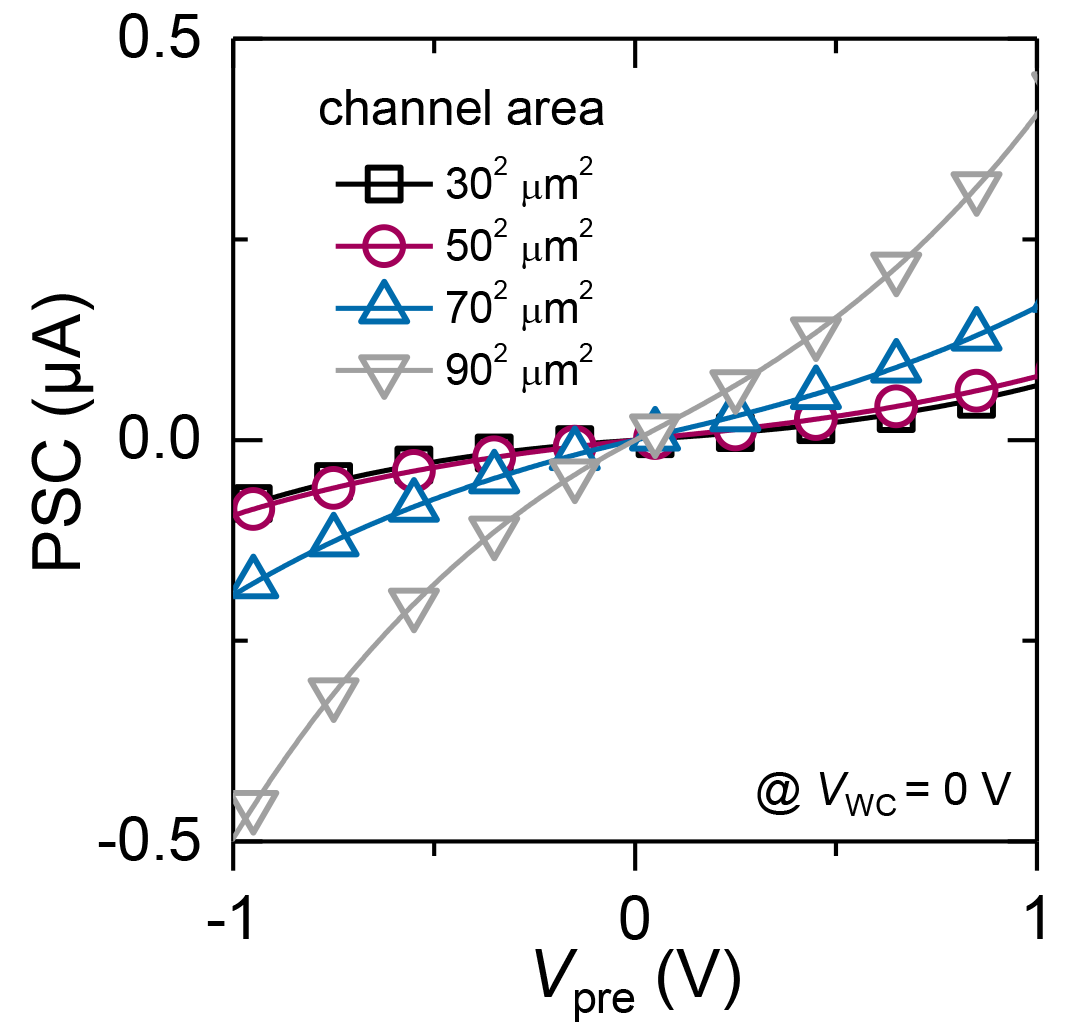


**Supplementary Figure 7 Channel area-dependent electrical property of vertical synapse.** Typical *I*_post_–*V*_pre_ characteristics of ion-gel-gated vertical P3HT synaptic devices with various channel areas in the absence of external voltage stimulus (*V*_WC_ = 0 V).


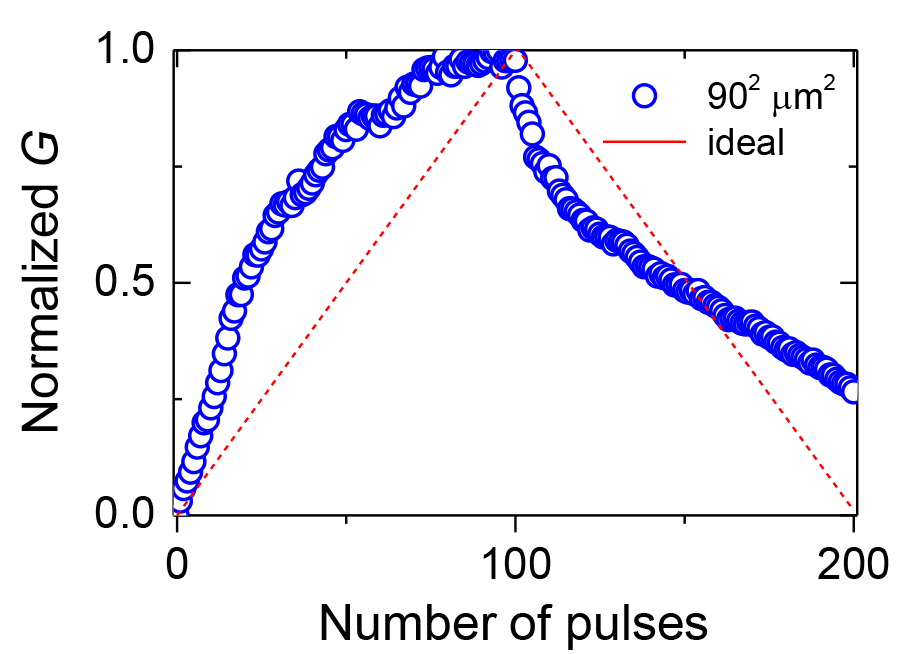


**Supplementary Figure 8** **Electrical property of vertical synapse.** Normalized LTP/D curves of synaptic device with largest channel area of 90 × 90 μm^2^.


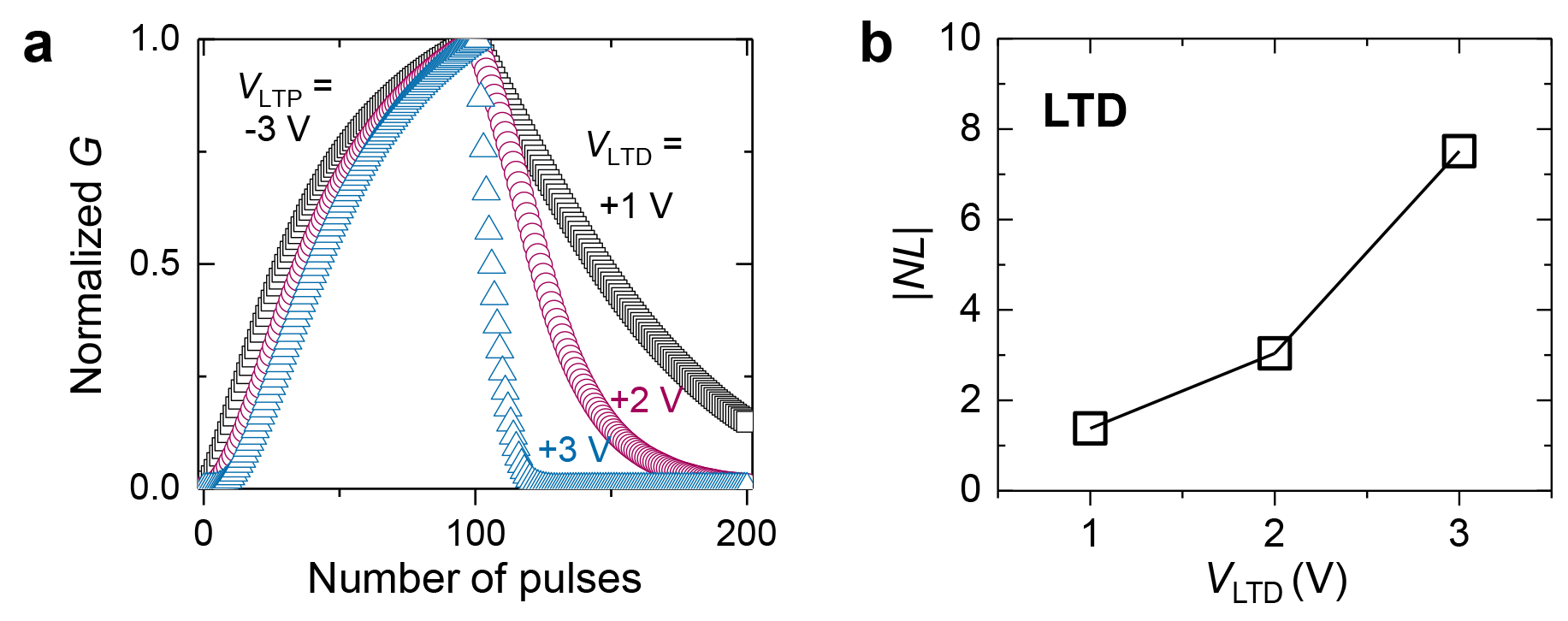


**Supplementary Figure 9** **LTD optimization of vertical synapse a**, LTP/D characteristic curves measured under depression pulses of +1, +2, and +3 V, where potentiation pulse was fixed at −3 V. **b**, |*NL|* extracted from LTP/D characteristic curves.


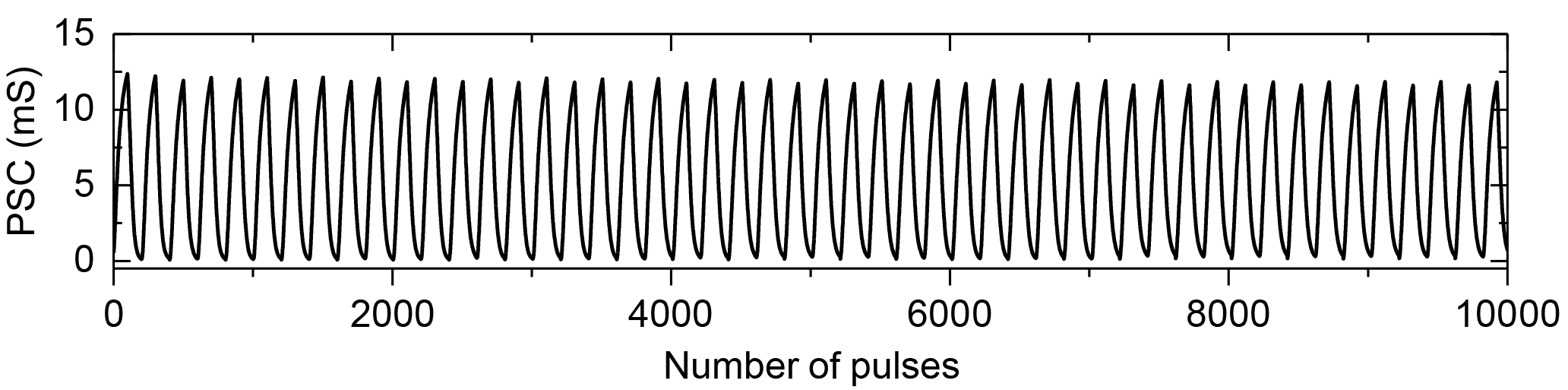


**Supplementary Figure10 Operational stability of vertical synapse.** PSC response of ion-gel-gated vertical P3HT synaptic device during test spanning 50 LTP/D cycles.


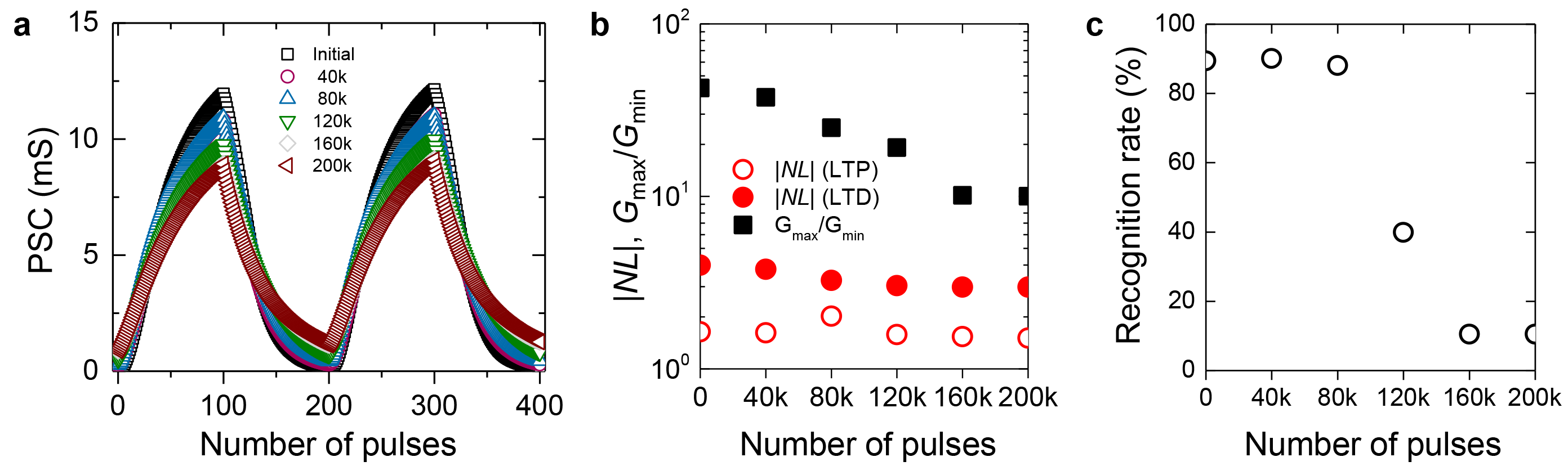


**Supplementary Figure 11** **Long-term stability of vertical synapse** **a**, PSC responses measured further to 200k pulses. **b**, |*NL|* and *G*_max_/*G*_min_ extracted at every 40k-th pulse from LTP/D characteristic curves. **c**, Recognition rate vs. number of pulses for artificial neural networks composed of parameters from (b).


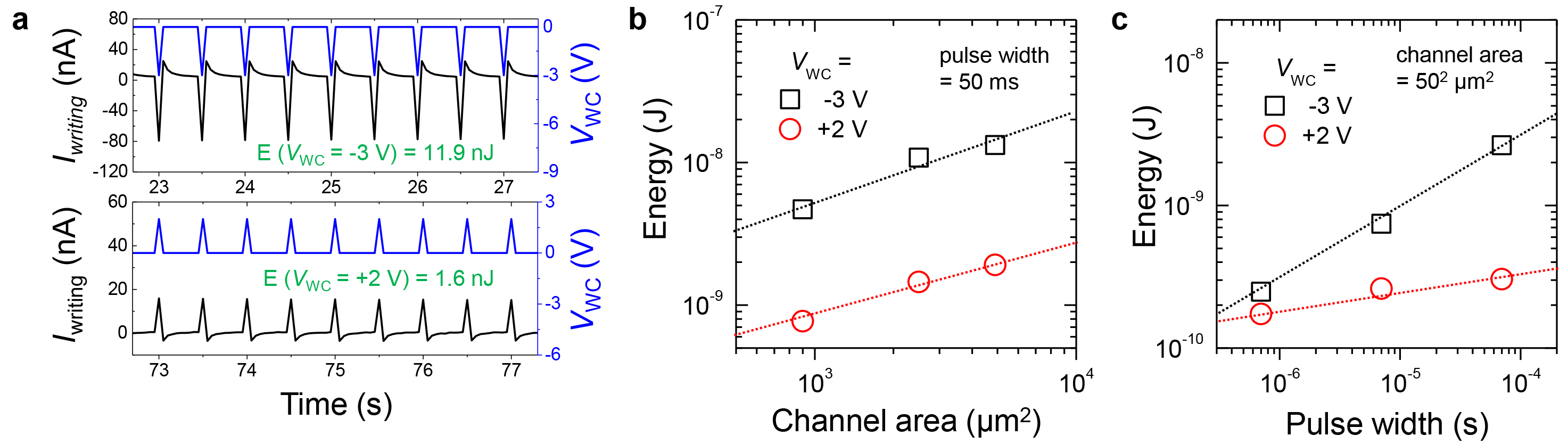


**Supplementary Figure 12** **Energy consumption of vertical synapse a**, Writing currents (*I*_writing_) measured when applying potentiation/depression pulses to weight-control terminal. **b-c**, Writing energies consumed by single potentiation/depression pulses as function of (b) channel area and (c) pulse width.


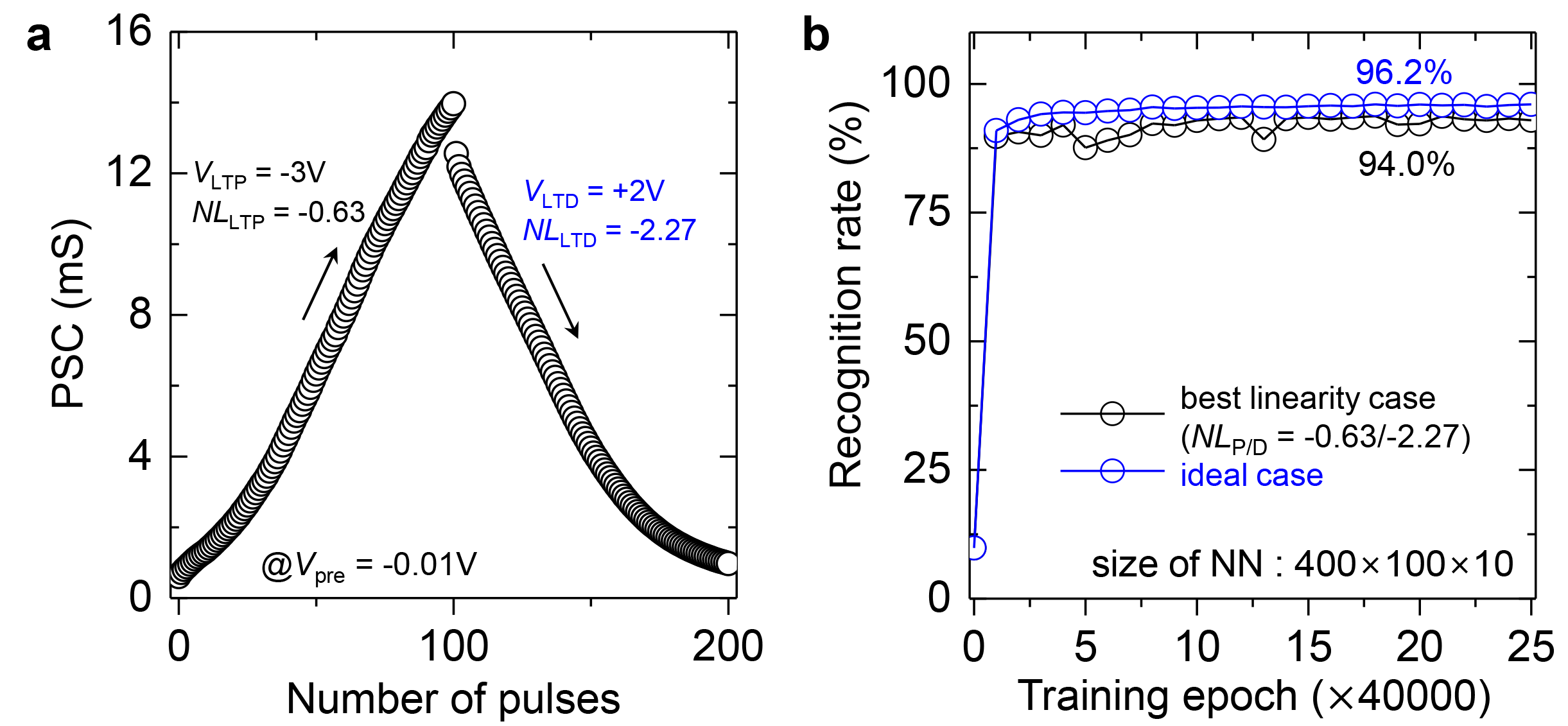


**Supplementary Figure 13 MNIST application of vertical synapse a**, LTP/D characteristic curve measured under *V*_LTP/D_ = −3 V/+2 V, where *NL*_LTP/D_ = −0.63/−2.27. **b**, Recognition rate vs. training epoch of best linearity case (black) and ideal case (blue).

**Supplementary Tables**

**Supplementary Table 1. Recent studies on the cycle-to-cycle variation and the corresponding recognition rate for MNIST digit images.**

|  | **This work** | AlO_x_/HfO_2_  RRAM^5^ | SiGe  epiRAM^6^ | *a*-MoO_3_  TFT^7^ | IGZO  FeFET^8^ |
| --- | --- | --- | --- | --- | --- |
| Cycle-to-cycle  variation | < 1% | 5% | < 1% | 6.5%/9.3%  (LTP/LTD) | 2.36% |
| Recognition rates (real/ideal devices) | 92.5%/96.2% | 41%/96% | 95.1%/97% | 87.3%/96.7% | 91.1%/94.1% |

**Supplementary Table 2. Maximum conductance and *NL* values extracted from LTP/LTD characteristic curves of 10 different synaptic devices.**

| **Device no.** | ***G*_max_ (mS)** | ***G*_min_ (mS)** | ***NL*_LTP_** | ***NL*_LTD_** |
| --- | --- | --- | --- | --- |
| 1 | 5.4 | 0.41 | -0.42 | -5.72 |
| 2 | 9.6 | 0.54 | 0.01 | -6.76 |
| 3 | 7.2 | 0.43 | -0.63 | -6.11 |
| 4 | 6.3 | 0.25 | 1.75 | -5.54 |
| 5 | 4.4 | 0.89 | -0.42 | -6.77 |
| 6 | 9.4 | 0.12 | -0.03 | -6.77 |
| 7 | 1.2 | 0.03 | -1.25 | -6.77 |
| 8 | 13.0 | 1.30 | -1.94 | -5.72 |
| 9 | 2.0 | 0.10 | -1.86 | -5.54 |
| 10 | 21.0 | 0.53 | -2.53 | -5.2 |

**Supplementary Notes**

**Supplementary Note 1. Penetration of ions into P3HT.**

The movement of anions (TFSI^-^ ions) under a negative *V*_WC_ was observed using scanning electron microscope-energy dispersive X-ray spectroscopy (SEM-EDS). A multilayered structure of P3HT/ion-gel/Au was fabricated on a highly *p*-doped Si wafer. We then applied a *V*_WC_ of −3 V for 5 s to the Au electrode to induce the penetration of anions (TFSI^-^) into the P3HT film. Finally, after removing the ion-gel/Au layer through a physical peel-off method, the cross section of the P3HT layer was investigated *via* SEM-EDS, as shown in **Supplementary Figure 1a–c**. Elemental signals for fluorine were detected to a depth of 100 nm from the P3HT surface, indicating that the TFSI^-^ mobile ions penetrated well into the P3HT layer under a negative *V*_WC_.

**Supplementary Note 2.** **Thickness-dependent electrical property of vertical synapse.**

To investigate the state decaying effects on the neural network training, we measured the LTP/D characteristics under pulse frequencies of 2, 4, 6, and 10 Hz, and then conducted the training/recognition tasks for MNIST patterns. The pulse amplitudes were fixed at −3 V/+2 V for potentiation/depression, respectively. As the pulse frequency varied from 2 to 10 Hz, the dynamic range increased from 20.9 to 39.6, and the linearity degraded from 0.72/−5.04 to 0.78/−5.2, respectively (**Supplementary Figure 4a**). Owing to the trade-off relationship between the dynamic range and linearity with respect to the pulse frequency, the recognition rate stayed in the range of 90 to 91% (**Supplementary Figure 4b**).

**Supplementary Note 3. Pulse width-dependent electrical property of vertical synapse**

To investigate the sub-microsecond writing capability, we applied a *V*_WC_s with three pulse widths of 300, 500, and 700 ns to the weight-control terminal, and then analyzed the PSC responses (**Supplementary Figure 5**). The pulse amplitudes were fixed at −3 V/+2 V for potentiation/depression, respectively. As shown in **Supplementary Figure 5a**, our device exhibited clear weight-updating behaviors of potentiation/depression for all cases of the pulse widths.

The LTP/D characteristics showed a relatively small dynamic range and high nonlinearity when compared to the values for the previous slow-speed write operation (**Supplementary Figure 5b**). It seems that only a few ions penetrated the P3HT channel region owing to their slow speed under high-speed operations, consequently degrading the channel controllability. Therefore, we believe that device scaling is a good solution for improving the channel controllability under sub-microsecond write/read operations.

**Supplementary Note 4. *NL* analysis of LTP and LTP characteristic curves**

There are several methods to evaluate the *NL* of the LTP/LTD characteristic curve^1-4^. Among them, we chose a method to tune *A*_P_ and *A*_D_ for finding the *G*_LTP_/*G*_LTD_ curves best matched to the measured LTP/LTD curves^3,4^. The *G*_LTP_/*G*_LTD_ curve model with the number of pulses (*P*) is represented as the following equations:

*G*_LTP_ = *B*∙(1 - exp(-*P*/*A*_P_)) + *G*_min_, 1

*G*_LTD_ = -*B*∙(1 - exp((*P* - *P*_max_)/*A*_D_)) + *G*_max_, 2

*B* = (*G*_max_ - *G*_min_)/(1 - exp(-*P*_max_/*A*_P,D_)) 3

where *G*_LTP_ and *G*_LTD_ are the conductance values for LTP and LTD, respectively. *G*_max_, *G*_min_, and *P*_max_ are the measured data that represent the maximum conductance, minimum conductance, and maximum pulse number, respectively. *B* is a fitting constant to normalize the conductance range. *A*_P_ and *A*_D_ are parameters that determine the nonlinearities of the weight update in the LTP and LTD regions, which are directly related to the *NL* values^3^.

The *G*_LTP_/*G*_LTD_ curves with respect to the *NL* ranging from 0 to 5 are displayed in **Supplementary Figure 6a**. By adjusting the *A*_P_ and *A*_D_ values, the *G*_LTP_/*G*_LTD_ curves are fitted to the measured LTP/LTD curves, and accordingly the *NL* values are determined (**Supplementary Figure 6b**).

**Supplementary Note 5.** **LTD optimization of vertical synapse**

To further investigate the symmetricity of the LTP/D characteristic curves, we applied depression pulses of +1, +2, and +3 V to the weight-control terminal, and then extracted the |*NL*|s for the LTP/D regions (**Supplementary Figure 9**). The potentiation pulse was fixed at −3 V. As a result, the LTP/D characteristic curve under a depression pulse of +2 V exhibited the best symmetricity. By contrast, a depression pulse of +1 V was not high enough to pull the TFSI^-^ ions from the P3HT channel, and thus the PSC did not recover to the initial state. A *V*_WC_ of +3 V was sufficiently high to return the PSC level to its initial level quickly, consequently showing very asymmetric LTP/D characteristics.

**Supplementary Note 6.** **Long-term stability of vertical synapse**

We fabricated new devices and then extracted the *NL*s and *G*_max_/*G*_min_ values at every 40k-th pulse from the LTP/D characteristics measured further to 200k pulses (**Supplementary Figure 11a** and **11b**). As a result, the |*NL|* values were almost unchanged for 200k pulses, and *G*_max_/*G*_min_ degraded below 10 after applying 120k pulses. The corresponding simulation results showed that the training/recognition performance was maintained perfectly up to 80k pulses (**Supplementary Figure 11**). This is probably owing to the stability of the |*NL|* values despite the degradation of *G*_max_/*G*_min_.

**Supplementary Note 7. Recent studies on the cycle-to-cycle variation and the corresponding recognition rate for MNIST digit images**

We summarized recent papers studying how the cycle-to-cycle variation of the LTP/LTD characteristics affects the recognition rate for MNIST digit patterns as shown in **Supplementary Table 1**. When the variation was under 3%^6,8^, the corresponding recognition rates were higher than 90%. On the contrary, for a high variation above 3%^5,7^, the recognition rate was seriously degraded below 90%.

Compared to these devices, our vertical organic synapse showed a very low cycle-to-cycle variation of less than 1% and thereby a high recognition rate of 92.5%. From the perspective of cycle-to-cycle variation, it is evident that the proposed device is acceptable for implementing a hardware neural network.

**Supplementary Note 8.** **Energy consumption of vertical synapse**

We calculated an approximate writing energy using the equation below.

*E*_writing_ = *V*_amp_ × *I*_peak_ × *t*_WC_ 4

where *V*_amp_ and *I*_peak_ represent the amplitude of the weight-control voltage (*V*_WC_) and the peak value of the writing current, respectively. *t*_WC_ is the pulse width of the *V*_WC_. As shown in **Supplementary Figure 12a**, the writing energy (*E*_writing_) for the potentiation pulse was −3 V × −79.1 nA × 50 ms = 11.9 nJ, and the *E*_writing_ for the depression pulse was 2 V × 15.9 nA × 50 ms = 1.6 nJ. Such values are higher than the energies below 10 pJ published in recent works, and thereby, it is required to be reduced further to be applied for hardware neural networks.

A possible method to reduce the *E*_writing_ is to scale down a channel area. To investigate the relationship between the *E*_writing_ and the channel area, we experimentally extracted the *E*_writing_ values for the potentiation/depression pulses when the channel areas were 30^2^, 50^2^, and 70^2^ μm^2^, as shown in **Supplementary Figure 12b**. The writing energies for the potentiation/depression pulses decreased from 13.3/1.9 nJ to 4.6/0.77 nJ, respectively, the channel area was scaled down. Furthermore, another possibility to reduce the *E*_writing_ was confirmed through pulse width engineering (**Supplementary Figure 12c**). When the pulse width was scaled down to 70 μs, the writing energies for the potentiation/depression pulses decreased to 2.6/0.3 nJ. These energies were reduced further to 0.25/0.17 nJ under the pulse width of 700 ns. Based on these experimental results, the writing energy of tens of pJ is expected to be achieved by scaling down the channel area to sub-5^2^ μm^2^ and reducing the pulse width to 500 ns.

**Supplementary Note 9. MNIST application of vertical synapse**

To further enhance the accuracy of the MNIST training, *NL* in the LTD region needed to be improved. For this, we reduced the amplitude of the depression pulses from +3 V to +2 V and investigated the LTP/D characteristics again. As shown in **Supplementary Figure 13a**, *NL* of LTD was improved to −2.27. Furthermore, owing to this improvement, a maximum accuracy of 94% could be achieved (**Supplementary Figure 13b**). Here, the size of the simulated NN was 400 × 100 × 10, and its maximum accuracy (ideal case) was 96.2%.

**Supplementary References**

1. Yang, C. S. et al. All-solid state synaptic transistor with ultralow conductance for neuromorphic computing. *Adv. Funct. Mater*. **28**, 1804170 (2018).

2. Yin, L. et al. Synaptic silicon-nanocrystal phototransistors for neuromorphic computing. *Nano Energy* **63**, 103859 (2019).

3. Chen, P., Peng, X. & Yu, S. Neurosim+: An integrated device-to-algorithm framework for benchmarking synaptic devices and array architectures. In: *2017 IEEE International Electron Devices Meeting (IEDM)* 6.1.1-6.1.4 (2017).

4. Qian, C. et al. Solar-stimulated optoelectronic synapse based on organic heterojunction with linearly potentiated synaptic weight for neuromorphic computing. *Nano Energy* **66**, 104095 (2019).

5. Woo, J. et al. Improved synaptic behavior under identical pulses using AlO_x_/HfO_2_ bilayer RRAM array for neuromorphic systems. *IEEE Electron Device Lett.* **37**, 994–997 (2016).

6. Choi, S. et al. SiGe epitaxial memory for neuromorphic computing with reproducible high performance based on engineered dislocations. *Nat. Mater*. **17**, 335–340 (2018).

7. Yang, C. S. et al. All-solid state synaptic transistor with ultralow conductance for neuromorphic computing. *Adv. Funct. Mater*. **28**, 1804170 (2018).

8. Kim, M. K. & Lee, J. S. Ferroelectric Analog Synaptic Transistors. *Nano Lett.* **19**, 2044–2050 (2019).
